# Junin Virus Activates p38 MAPK and HSP27 Upon Entry

**DOI:** 10.3389/fcimb.2022.798978

**Published:** 2022-04-07

**Authors:** Collin J. Fitzpatrick, Rajini R. Mudhasani, Louis A. Altamura, Catherine E. Campbell, Julie P. Tran, Brett F. Beitzel, Aarthi Narayanan, Cynthia L. de la Fuente, Kylene Kehn-Hall, Jeffrey M. Smith, Connie S. Schmaljohn, Aura R. Garrison

**Affiliations:** ^1^ United States Army Medical Research Institute of Infectious Diseases, Fort Detrick, MD, United States; ^2^ DCE Consulting, Vienna, VA, United States; ^3^ National Center for Biodefense and Infectious Diseases, George Mason University, Manassas, VA, United States

**Keywords:** Junin virus, cellular pathways, HSP27, p38 MAPK, antiviral

## Abstract

Junín virus (JUNV), a New World arenavirus, is a rodent-borne virus and the causative agent of Argentine hemorrhagic fever. Humans become infected through exposure to rodent host secreta and excreta and the resulting infection can lead to an acute inflammatory disease with significant morbidity and mortality. Little is understood about the molecular pathogenesis of arenavirus hemorrhagic fever infections. We utilized Reverse Phase Protein Microarrays (RPPA) to compare global alterations in the host proteome following infection with an attenuated vaccine strain, Candid#1 (CD1), and the most parental virulent strain, XJ13, of JUNV in a human cell culture line. Human small airway epithelial cells were infected with CD1 or XJ13 at an MOI of 10, or mock infected. To determine proteomic changes at early timepoints (T = 1, 3, 8 and 24 h), the JUNV infected or mock infected cells were lysed in compatible buffers for RPPA. Out of 113 proteins that were examined by RPPA, 14 proteins were significantly altered following JUNV infection. Several proteins were commonly phosphorylated between the two strains and these correspond to entry and early replication events, to include p38 mitogen-activated protein kinase (MAPK), heat shock protein 27 (HSP27), and nuclear factor kappa B (NFκB). We qualitatively confirmed the alterations of these three proteins following infection by western blot analysis. We also determined that the inhibition of either p38 MAPK, with the small molecule inhibitor SB 203580 or siRNA knockdown, or HSP27, by siRNA knockdown, significantly decreases JUNV replication. Our data suggests that HSP27 phosphorylation at S82 upon virus infection is dependent on p38 MAPK activity. This work sheds light on the nuances of arenavirus replication.

## Introduction

Junín virus (JUNV) is a single stranded RNA virus and the causative agent of Argentine hemorrhagic fever (AHF). Humans become infected following exposure to rodent host secreta and excreta and the resulting infection can lead to an acute inflammatory disease, which in the absence of treatment has a case fatality rate approaching 30% (Gomez et al., 2011). JUNV is endemic to the Pampas of north-central Argentina, and infection is most common in farm workers who come in contact with excreta of the rodent host, *Calomys musculinus*, during harvest periods (Goni et al., 2010). AHF is a prototypical HF, with acute cases presenting with gross manifestations (petechiae, ecchymoses and injected conjunctiva) and clinical markers (hypotension, leucopenia, thrombocytopenia, and proteinurea) indicative of vascular damage. Neurological dysfunction, coma and convulsions are also observed in severe cases, and these are indicative of a poor prognosis. It is estimated that several hundred to thousands of infections occur yearly (Gomez et al., 2011). JUNV is highly infectious by the aerosol route, although human-to-human transmission is rare.

Viruses utilize host protein networks for all aspects of their replication cycle, and by characterizing changes in the host cell in response to infections we can extrapolate how these viruses reprogram cellular processes. Phosphorylation events are critical for understanding the cell-signaling events that occur during virus replication. A number of pathways are controlled by a series of modifications to proteins such as phosphorylation, acetylation, ubiquitination, and methylation, not just at the transcriptional or translational level of proteins. The comparison of the cellular modifications following infection with virulent and attenuated strains of viruses may also shed light on mechanisms behind viral attenuation. JUNV enters cells mediated by the viral glycoproteins (GPs), GP-1 and GP-2, and the cellular transferrin receptor 1 (TfR1) (Radoshitzky et al., 2007). Beyond entry there is little known about the interactions between the GPs and the host cell and the role in pathogenesis. In addition, the mechanism of attenuation of JUNV has not been determined. Albarino et al. found that the attenuation of strain Candid#1 (CD1) in mice appears to be determined by substitutions of the transmembrane domain of GP2, F427I (Albarino et al., 2011a). Droniou-Bonzom et al. used pseudoviruses to determine that the F427I substitutions in CD1 reduced infectivity of the virus *in vitro*, which may contribute to the attenuated phenotype (Droniou-Bonzom et al., 2011). Another group found that the loss of an N-linked glycosylation motif in CD1, compared to the less attenuated parental strain, leads to an aggregation of the glycoproteins in the endoplasmic reticulum (ER) (Manning et al., 2020). This aggregation contributed to an ER stress response, and increased lysosomal-mediated degradation of the aggregates. Although the growth properties of the virulent and attenuated strains do not differ *in vitro*, the authors suggest that the increase in degradation may limit the spread of the virus due to an increase in antigen presentation and a decrease in surface glycoproteins. Overall, there is a paucity of information regarding global changes in host pathways following attenuation and the potential impact on pathogenesis.

In this study, we used reverse phase protein microarrays (RPPA) to compare global alterations in the host proteome following JUNV infection with an attenuated vaccine strain, Candid#1 (CD1), or a virulent strain, XJ13, in a human cell culture line. There were common changes in proteins between both strains that corresponded to entry and early replication of the viruses, and divergent alterations of proteins associated with cellular anti-viral responses and apoptosis. Collectively, our findings shed some light on cellular processes involved in replication of JUNV, and changes in the anti-viral and cellular pro-survival responses of cells infected with an attenuated or virulent strain of JUNV. These results may provide insight into determining the mechanisms of the pathogenic differences between these strains.

## Methods

### Cell Culture

BSRT7/5 cells, a cell line derived from BHK-21 baby hamster kidney cells which stably expresses the T7 RNA polymerase gene, were a generous gift from César Albariño from the Center for Disease Control and Prevention (CDC) and were maintained in MEM (Corning) with 10% FBS (HyClone). Vero cells (ATCC) were maintained in complete Eagle’s minimum essential medium EMEM (Corning) supplemented with 1% L-glutamine (HyClone), 10% FBS (ThermoScientific/Hyclone), and 1% penicillin/streptomycin (Gibco). Human small airway epithelial cells (HSAECs) were acquired from Cambrex Inc., Walkersville, MD and maintained in Ham’s F-12 media (Lonza) with 10% FBS, 1% L-glutamine, 1% Sodium Pyruvate (Sigma), 1% penicillin/streptomycin, 1% non-essential amino acids (Gibco), and 0.1% 55mM beta-mercaptoethanol (Gibco). All cell lines were maintained at 37°C with 5% CO_2_. 293T cells, a human embryonic kidney cell line, were obtained from the ATCC and maintained in DMEM (Corning) with 10% FBS (HyClone). A549 cells, a human alveolar basal epithelial cell line, were obtained from the ATCC and maintained in Ham’s F-12 media (Lonza) with 10% FBS, 1% L-glutamine, 1% Sodium Pyruvate (Sigma), 1% penicillin/streptomycin, and 1% non-essential amino acids (Gibco).

### Virus and Pseudovirus Stocks

Plasmids for the rescue of recombinant JUNV strain XJ13 and CD1 were a generous gift from César Albariño (CDC). Rescue of JUNV XJ13 and Candid 1 was performed as previously described (Albarino et al., 2009). Briefly, 1 × 10^6^ BSRT7/5 cells per well in 6-well plates were transfected with a 1:3 ratio of TransIT LT1 (Mirius) to 2 µg of each of the two plasmids per well encoding full-length cDNA copies of the viral S (pJCd1S or pJXJ13S) and L antigenomic segments (pJCd1L or pJXJ13L). Transfected BSRT7/5 cell supernatants were harvested 3 days post-transfection and clarified by low-speed centrifugation. Both rescued viruses were titered by plaque assay on Vero cells. Vero cells with the media removed, approximately 80–90% confluent, in 6-well plates were infected with 200 µl of a dilution series, from 10^−1^ to 10^−8^, of the clarified supernatant from the virus rescues. The plates were incubated for 60 min at 37°C/5% CO_2_ and gently rocked every 15 min. Approximately 2 ml of a primary overlay was added to each well with a final concentration of 1× EBME, 10% FBS, 4% L-Glutamine, and 1% Penn/strep and 0.6% Seakem ME. After 3 days a secondary overlay of 5% Neutral Rd in Saline A was added. Plaques were counted on day 6 post infection. A second passage of JUNV was propagated by infecting Vero cells in T225 flasks with a multiplicity of infection (MOI) of 0.001. The supernatants were harvested and clarified 5 days post-infection and the titer was determined by plaque assay as described above. All JUNV work was performed in BSL-4 containment.

Pseudovirus for looking into arenavirus entry was produced with pseudotyped retroviral particles expressing Junin or Candid-1 glycoporotein (GP). The pseudovirus was produced from 293T cells by transfecting at 1:1:1 ratio of plasmids expressing murine leukemia virus (MLV) gag/pol, Junin GP or Candid-1 GP (a kind gift from Dr. Paula Cannon from the University of Southern California) and pQCXIX transduction vector (BD Biosciences) expressing enhanced green fluorescent protein (EGFP). Fugene HD (Promega) was used as a transfection reagent, by following the instructions from the manufacturer manual. Six hours post transfection, the media on the cells was replaced with fresh media. Two days post transfection, the supernatants were clarified to remove the cells by centrifugation at 1,000 rpm for 10 min, followed by freezing the pseudovirus containing supernatants as one milliliter aliquots at −80°C. For viral entry assays, we initially determined the optimal conditions to achieve a linear increase in transduction rates from 10 to 80% with increasing titers of pseudovirus in HSAEC cells. From this titration assay, the volume of pseudovirus required to achieve 50–60% transduction rates was determined and used for viral entry assays.

### HSAEC Infections and Sample Harvest

For optimization of JUNV infection of HSAEC cells, 1.4 × 10^4^ cells were seeded per cell in 96-well black µClear plates (Greiner Bio-One). Twenty-four hours post-seed the cells were infected with either JUNV strain XJ13 or strain CD1 from an MOI of 0–34 or 0–39 respectively, 3 wells per MOI. After 24 h the cells were fixed in 10% neutral buffered formalin for a minimum of 24 h.

For RPPA HSAEC cells (7.5 × 10^5^ cells/well) were seeded into 6-well plates. Infections with XJ13 and CD1 viruses were performed at an MOI 10. After a one-hour incubation, virus inoculum was removed, cells were washed three times with phosphate buffered saline (PBS; Gibco-Invitrogen) and cultured until harvest. Conditioned media was used for all infections and subsequent culturing to limit potential activation of signaling cascades due to fresh serum. Mock samples were treated similarly with conditioned media alone.

Two wells for each virus and the mock control were harvested at 1, 3, 8, and 24 hpi for biological replicates. Extracellular supernatants were clarified by centrifugation, aliquoted, and stored at −80°C. Cells were then washed once with PBS and lysed directly in blue lysis buffer which contains 1.25:1 ratio of 2× Novex^®^ Tris-Glycine SDS Sample Buffer and Tissue Protein Extraction Reagent (T-PER; ThermoFisher Scientific), 2.1 mM EDTA pH 8.0, 0.85 mM NaF, 170 μM Na_3_VO_4_, 27.7 mM dithiothreitol (DTT), and EDTA-free Complete Protease Inhibitor Cocktail tablet (Roche). For lysis the blue lysis buffer was added to cells directly in wells for 5 min with occasional rocking and the lysate was harvested. Samples were incubated at 95°C for 15 min to completely inactivate the virus. Samples were stored at −80°C until further processing or analysis. This experiment was performed three times for three technical replicates. All JUNV work was handled in a BSL-4 containment.

### RPPA

RPPA was performed as previously described (de la Fuente et al., 2018). Briefly, cell lysates were immobilized onto nitrocellulose coated slides (Grace Bio-Labs) using an Aushon 2470 arrayer (Aushon BioSystems). Each sample was printed in triplicate along with standard curves for internal quality control. Selected arrays were stained with Sypro Ruby Protein Blot Stain (Life Technologies) following manufacturing instructions in order to quantify the amount of protein present in each sample (Pin et al., 2014). The remaining arrays were treated for 15 min at room temperature with the mild stripping reagent, Reblot Antibody Stripping solution (EMD Millipore), in order to expose antigenic sites prior to antibody staining. The arrays were then washed twice for 5 min at room temperature in PBS (Life Technologies), and incubated for 5 h in I-Block (Applied Biosystems) in order to block non-specific binding sites on the nitrocellulose. Using a Dako Autostainer Universal Staining System, arrays are first probed with 3% hydrogen peroxide, biotin blocking system (Dako Cytomation), and an additional serum free protein block (Dako Cytomation) to reduce non-specific binding between endogenous proteins and the detection system.

Arrays were probed with 113 antibodies, of which 90 were phospho-specific. A complete list of all of the antibodies used was previously described by de la Fuente et al. (2018). The majority of the antibodies were purchased from the Cell Signaling Technologies (CST), while the following were purchased from other vendors: Beclin 1 (#PRS3613, Sigma), Histone H3 S10 and S28 (#06-570 and 07-145, Upstate Biotechnology), IL10 (#ab52909, Abcam), IL6 (#5143-100, BioVision), IL8 (#ab7747, Abcam), MMP-11 (#ab52904, Abcam), p27 T187 (#71-7700, Zymed), PDGFR Y716 (#07-021, Upstate Biotechnology), PKC alpha S657 (#06-822, Upstate Biotechnology), Protein Phosphatase 1 beta (#ab53315, Abcam), Ron Y1353 (#5176-1, Epitomics), and S100A7 Calcium BP (#H00006278-A01, Abnova). Antibodies were validated for their use on the array by Western Blot to determine antibody specificity. Only antibodies showing a single band at the expected molecular weight were used on the arrays (Signore et al., 2017). Biotinylated anti-rabbit (Vector Laboratories, Inc.) or anti-mouse secondary antibody (CSA; Dako Cytomation) was used in conjunction with GenPoint™ kit (Dako Cytomation), a commercially available tyramide-based signal amplification system. Fluorescent detection was obtained through the use of IRDye 680RD Streptavidin (LI-COR Biosciences) according to the recommendations of the manufacturer. Antibody and Sypro Ruby stained slides were scanned on a Tecan laser scanner (TECAN) using the 620 and 580 nm weight length channel, respectively.

Images were analyzed with MicroVigene Software Version 5.1.0.0 (Vigenetech). The software performs spot finding along with subtraction of the local background and non-specific binding generated by the secondary antibody. Each sample signal was normalized to the corresponding amount of protein derived from the Sypro Ruby stained slides and triplicates were averaged. All RPPA data was annotated with Uniprot IDs and total protein or phospho-protein was noted. The average fold changes and t-test was calculated for the matched triplicate samples for each JUNV-infected/mock comparison per timepoint. A t-test was also performed on the triplicate samples for each infected/mock set per timepoint.

### Western Blots

Blue lysis samples were thawed and boiled for 5 min with the addition of 10% BME. Lysates were then loaded on a 4–12% Bis-Tris Protein mini gel (1.0 mm; Life Technologies) and the separated proteins were transferred to a polyvinylidene difluoride (PVDF) membrane. After blocking in Odyssey TBS buffer (Li-COR) blots were incubated overnight at 4°C with primary antibodies against total p38MAPK (Cell Signaling), p38 MAPK T180/Y182 (Cell Signaling), COX IV (Li-Cor), total HSP 27 (Cell Signaling), HSP27 S82 (Cell Signaling), and GAPDH (Santa Cruz). The bound primary antibody was then detected with the appropriate secondary antibodies, IR680 or IR800 conjugated goat anti-mouse or goat anti-rabbit IgG (Li-COR). Blots were imaged on the Li-COR Odyssey CLx and images were analyzed utilizing Li-COR Image Studio Ver 5.2 software. The data was analyzed by two-way ANOVA for multiple comparisons in GraphPad Prism.

### SB 203580 p38 MAPK Viral Inhibition

A 25 mM stock of SB 203580 (Abcam) in DMSO was diluted in Ham’s F-12 media for a 2× dose curve from 20 to 80 µM. HSAEC cells in 96-well black µClear plates (Greiner Bio-One) were pre-treated in triplicate for 2 h at 37°C/5% CO_2_ with the dose curve of SB 203580, final concentration on cells of 10 to 40 µM, or a DMSO concentration equivalent control for each concentration of the compound. JUNV CD1 or Romero pseudovirus was added to the wells at an MOI of 1 or at a concentration to achieve 50–60% transduction respectively, and incubated at 37°C/5% CO_2_ for 2 h in the presence of the compound. After the incubation the inoculum with the compound of DMSO control was replaced and incubated for 24 h at 37°C/5% CO_2_. The cells were fixed in 10% buffered formalin for 1 h and stained for high content imaging analysis. The data was analyzed by two-way ANOVA for multiple comparisons in GraphPad Prism.

To examine if phosphorylation of HSP27 following JUNV infection is downstream of p38 MAPK, HSAEC cells seeded in 6-well plates (Corning) were pretreated with 40 nM SB 203580, or the equivalent concentration of DMSO, or media alone for 2 h in duplicate. The cells were then infected with CD1 or mock infected (media only). At 0.5–24 h the cells were lysed, and the duplicate wells were pooled and probed for HSP27 S82 by Western blot.

### siRNA Transfections

For siRNA experiments, in duplicate plates HSAECs seeded in 96-well plates (2 × 10^4^ cells/well) were transfected with media alone (Mock) or siRNAs against non-targeting control (Negative control #2, Ambion), MAPK14 (s3585, Ambion), and HSPB1(s194537, Ambion). All siRNA transfections were performed with Dharmafect 1 and 10, 25, or 50 nM of each siRNA in triplicate. After 24 hpt, transfection media was removed and fresh complete media was added. At 48 hpt, protein lysates were collected from one plate, three wells per sample, and analyzed for protein expression. The second plate was infected with JUNV Candid#1 at an MOI 1, and fixed in 10% neutral buffered formalin 24 h post infection and stained for high content imaging analysis. The data was analyzed by two-way ANOVA for multiple comparisons in GraphPad Prism.

### High Content Imaging

For infection optimization high-content imaging (HCI) experiments, JUNV nucleocapsid (N) protein was detected using the mouse monoclonal antibody QB-06-AE05 (BEI Resources). The cells were permeabilized using 0.1% Triton-x 100 (Sigma-Aldrich) for 10 min at room temperature, then the cells were washed twice in PBS. Non-specific epitopes were bound by incubating the fixed cells with a blocking solution containing 3% bovine serum albumin in PBS (Sigma-Aldrich) at room temperature for 30–60 min. The primary antibody for JUNV, mouse monoclonal QB-06-AE05, was diluted to 2.2 µg/ml in blocking buffer, and 50 µl was added to each well and incubated for 1 h. The cells were then washed three times in PBS. An AlexaFluor 488-conjugated goat anti-mouse secondary antibody (Invitrogen) was used for JUNV detection. The secondary antibody was added to each well at a concentration of 2 µg/ml and the plates were incubated at room temperature for 1 h, then the cells were washed three times in PBS. The cell nuclei were stained with 1 µg/ml of Hoechst 33342 stain (Invitrogen), and the plasma membranes were stained with 500 ng/ml of CellMask Deep Red (Invitrogen). High-content data were acquired and analyzed on an Operetta (PerkinElmer), high-throughput, wide-field fluorescence microscope reader (Perkin Elmer, Waltham, MA 02451) using the non-confocal setting with a ×10 objective. Image analysis was performed on the fly by using the Harmony 3.0 software package (PerkinElemer). For SB 203580 inhibition experiments with JUNV CD 1 the cell were stained as above for N protein, and for pseudovirus detection the GFP tag was utilized. Hoechst and CellMask Deep Red was used as described above. All imaging for the inhibition studies was performed and analyzed on the Lionheart FX (BioTek) and Software Version 3.03.14. All quantifications are based on data obtained from triplicate samples. Error bars in all figures indicate standard deviations. P-values were calculated using an unpaired Student’s t-test. P-values below 0.05 were considered significant.

## Results

### RPPA Reveals Common Early Increased Phosphorylation of NFκB, p38 MAPK, and HSP27

The viruses used for all experiments were generated utilizing reverse genetics systems developed by Albarino et al. to minimize potential variability in results due to non-clonal virus stocks (Albarino et al., 2009). First, we determined the multiplicity of infection (MOI) of both JUNV strain XJ13 and CD1 that infect approximately 50% of human small airway epithelial cells (HSAEC), a primary immortalized cell line, as measured by high content imaging 24-hour post infection. Several groups have published that both strains of JUNV replicate with similar kinetics in several cell lines (Albarino et al., 2009; Albarino et al., 2011b; Manning et al., 2020). We determined that the replication kinetics, as measured by JUNV positivity by HCI, was similar in HSAEC cells as well, and the optimal MOI for approximately 50% infection was determined to be between an MOI of 9–10 for both strains (**Supplementary Figure 1**). HSAECs were infected with CD1 or XJ13 at an MOI of 10, or mock infected with conditioned media. One and three-hour timepoints post infection were chosen to represent early stages of viral entry and replication, the eight and 24-hour timepoints were selected for mid to late stages of a single round of viral replication. Duplicate samples of JUNV infected or mock infected HSAEC cells were lysed in a buffer compatible with RPPA at each post infection timepoint, in three separate experiments.

Out of 113 proteins that were examined by RPPA, the phosphorylation status of 14 proteins was significantly altered following JUNV infection (**Table 1** and **Supplementary Table 1** **)**. Fold changes were determined by comparing the mock infected to the JUNV infected signal at each timepoint, and a t-test was calculated to determine if the fold-change was significant across all three experiments. At the one-hour timepoint there was an increase in phospho-signaling of three proteins HSP27 (S82), p38 MAPK (T180/Y182), and NFkB p65 (S536), upon infection with both JUNV strains. One protein, AKT, had a decrease in phosphorylation at T308 in both strains at the 24-hour timepoint, and an increase in phospho-signaling in XJ13 at the 8-hour but not CD1, however the S473 phosphorylation site was not altered with either strain. HSP27 activates actin polymerization and reorganization, which may result from virus entry and transport within the cell during the early stages of virus replication, and JUNV entry appears to be dependent on an intact actin network (Martinez et al., 2008).

**Table 1:** Significant changes in signaling protein phosphorylation following infection with JUNV.

	Description		Candid#1 versus Mock				XJ13 versus mock			
		Unitprot	1h	3h	8h	24h	1h	3h	8h	24h
Entry Shared	NFkB p65 S536	Q04206	1.93	1.04	0.89	1.05	1.55	1.15	1.06	0.97
	p38 MAPK T180/Y182	Q16539	1.95	0.87	0.84	0.89	1.38	0.94	0.97	0.91
	HSP27 S82	P04792	2.09	0.76	0.69	1.23	1.48	1.11	0.94	0.94
Entry Unique to XJ13	p90 RSK T359/S363	Q15418	0.93	0.89	0.89	1.02	0.83	0.88	0.91	0.99
	A_Raf S299	P10398	1.11	0.56	0.75	3.91	0.75	0.82	0.89	3.74
	S100 Calcium Binding Protein A7	P31151	1.00	0.88	0.91	0.93	0.82	0.85	0.91	0.92
Shared	Akt T308	P317549	1.05	1.12	0.86	0.67	0.99	1.01	1.24	0.53
Unique to XJ13	PKC delta T505	Q05655	1.02	1.03	0.92	0.85	0.94	0.98	1.21	0.75
	ASK1 S83	Q99683	1.04	0.89	0.86	0.72	0.91	0.92	1.39	0.57
	MPO	P05164	1.30	0.82	0.76	0.58	0.86	0.82	1.35	0.13
Unique to CD1	Beta Catenin S33/37/T41	P35222	1.01	0.99	0.90	0.83	0.98	1.00	1.00	0.89
	Smad1 (Ser463/465)/ Smad5 (Ser463/465)/ Smad9 (Ser465/467)	Q15797	1.01	0.97	0.85	1.41	1.15	1.06	1.16	1.23
	SOCS1	O15524	1.34	0.83	0.86	0.65	1.08	0.85	1.14	0.81
	PKCζ/λ T410/403	P05129	1.02	1.24	0.92	0.91	1.06	1.20	1.25	1.10

RPPA results for JUNV XJ13 (XJ) and Candid 1 (CD1) compared to mock infected cells. Changes highlighted in yellow (decreased phosphorylation) or blue (increased phosphorylation) have at least a 1.2 fold change and a p ≤0.1 as measured by a t-test.

Only one other protein had an increase in phosphorylation at an early timepoint (3 h), protein kinase C zeta lambda (PKCζ/λ), which was phosphorylated at T410 (ζ) and T403 (λ) and was unique to CD1. Other proteins that were altered at 1 h, with a significant decrease in phopho-signaling or total protein were p90 RSK (T359/S363), A-Raf (S299), and S100A7 Calcium BP. Six proteins were altered in phospho-signaling at the mid and late timepoints: PKCΔ (T505), ASK1 (S83), Smad1 (Ser463/465)/Smad5 (Ser463/465)/Smad9 (Ser465/467), Beta Catenin (S33/37/T41), Myloperoxidase (MPO), and SOCS1.

**Figure 1:**
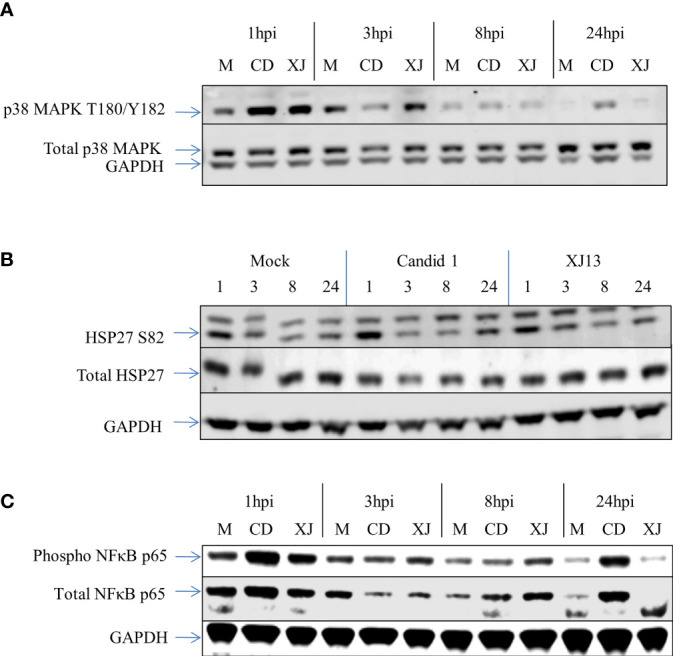
Western blot data for p38 MAPK, HSP-27, and NFκB from RPPA samples. HSAEC cells were infected with an MOI of 10 for each virus, or mock infected. At 1, 3, 8, or 24 h post infection the cells were lysed and the virus was inactivated. The Western blot was performed with one sample per virus or mock per timepoint. The RPPA data was performed and analyzed utilizing technical duplicates and experimental triplicates, for a total of 6 samples per timepoint per virus or mock. **(A)** A Western blot representative of three separate experiments for p38 MAPK. **(B)** A Western blot representative of three separate experiments for HSP-27. **(C)** A Western blot representative of three separate experiments for NFκB.

Western Blot analysis was then used to qualitatively evaluate some of the altered proteins identified by RPPA (**Figures 1A–C**). We confirmed the increase in phosphorylation of p38 MAPK at T180/Y182 in both strains (**Figure 1A**) at the one-hour timepoint, the Western blot image shown is representative of 3 sample sets that were analyzed from the three independent experiments utilized for RPPA. In **Figure 1A** one representative Western blot image of one of the three independent experiments is shown. The increase in phosphorylated p38 MAPK at 1 h was evident in all three Western blots for CD1 and XJ13, and the increase appears slightly greater in CD1 in comparison to XJ13 at the one-hour timepoint. This difference in the level of increase between the strains was consistent with the RPPA data in **Table 1**, with a 1.95 fold difference in CD1 over mock and a 1.38 fold difference in XJ13 over mock. The marginal increase in phosphorylation in p38 MAPK in the CD1 sample at 24 h in **Figure 1A** was not present in the Western blot of the other two independent experiments, thus the 24-hour timepoint activation of p38 MAPK following JUNV infection was inconsistent and may depend on the timing of virus release and entry into uninfected cells in the sample wells.

The increase in phosphorylation of HSP27 at S82 as indicated by RPPA was also visualized by Western Blot analysis (**Figure 1B**). A representative Western Blot for HSP27 S82 is shown. There was a visible increase in HSP27 S82 at the 1-hour timepoint across all of the Western blots with CD1. One of the three blots did not show an increase in XJ13 at the 1-hour timepoint when normalized to GAPDH, but as only one of the technical replicates from the 3 independent experiments were analyzed by Western blot, inherent variability in samples may be more evident by Western blot than RPPA. Consistent with the with p38 MAPK data in **Figure 1A**, the level of increase in phosphorylation of HSP27 appears greater in CD1 in comparison to XJ13 in **Figure 1B**. This slight difference between the strains is also consistent with the RPPA data in **Table 1**, with a 2.09 fold increase in CD1 over mock and a 1.48 fold increase in XJ13 over mock. Based on the size of the top migrating band shown in the HSP27 S82 blot (**Figure 1B**) this may be the triple phosphorylated form of HSP27 or it may be a non-specific background band, as the top band was not present in the other two HSP-27 S82 Western blots. The increased phosphorylation of NFκB p65 for both strains at the one-hour timepoint was also visualized by Western blot (**Figure 1C**). All three blots show an increase in phosphorylated NFκB with both viruses at 1-hour post infection, and one representative blot is shown. Again, the increase in phosphorylation in NFκB p65 upon CD1 infection was greater than XJ13 by RPPA (**Table 1**), 1.93 fold increase over mock and 1.55 fold increase over mock respectively, and appeared greater in the Western blot.

### Inhibition of p38 MAPK Activity Reduces JUNV Infection Induced HSP27 Phosphorylation

**Figure 2:**
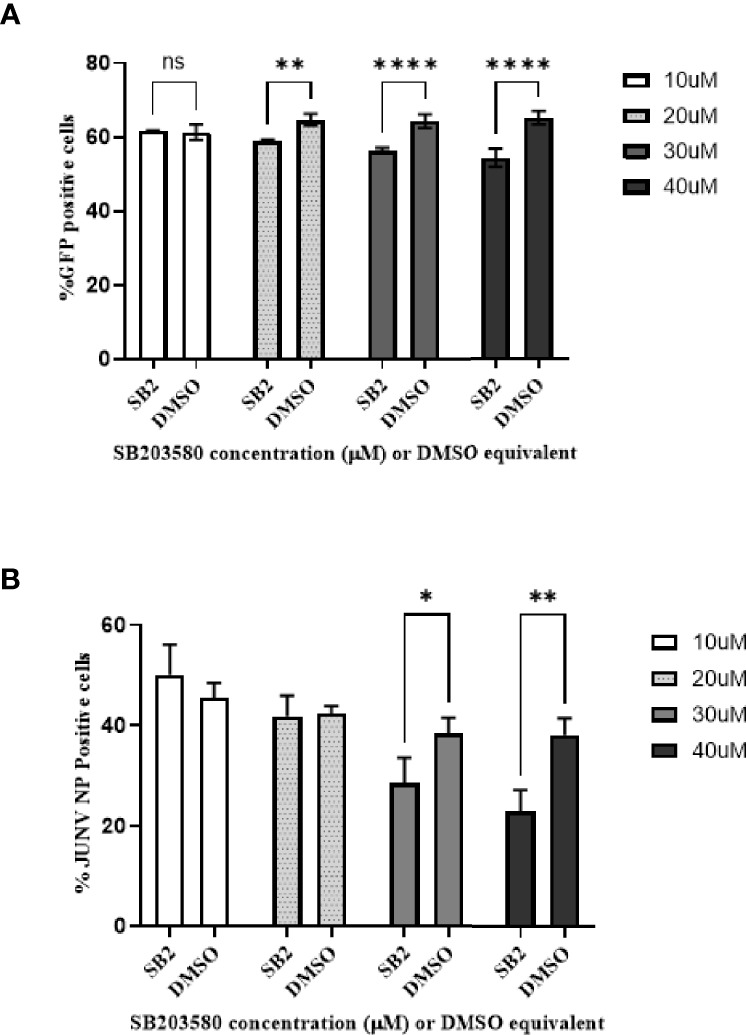
Phospho-p38 MAPK inhibitor decreases JUNV pseudovirus and wild type virus replication *in vitro* in a dose–response. HSAEC cells were pretreated with SB 203580 in a dose curve for 2 h in triplicate wells, and then infected with JUNV Romero pseudovirus **(A)** which contains a GFP signal or JUNV CD1 **(B)**. **(A)** The pseudovirus GFP signal was measured by high-content imaging 48-hours post infection. **(B)** For the JUNV CD1 samples, 24-hours post infection the cells were fixed and the JUNV nucleocapsid protein was stained with a mouse monoclonal anti-N antibody and detected with an Alexafluor-488 conjugated secondary, the nuclei was stained with Hoescht and the signal was measured by high content imaging. The data is plotted as means with standard deviations. P-values less than 0.05 were considered significant. One representative of two experiments is shown for both the pseudovirus and wild type virus. **(A)** **p=0.0018, ****p<0.0001, ns, not significant. **(B)** *p=0.0344, **p=0.0011.

Next, we sought to determine if phosphorylated p38 MAPK activity plays a role in virus replication. SB 203580 is an inhibitor of phosphorylated p38 MAPK activity as it prevents the interaction of phosphorylated p38 MAPK with downstream effectors, such as HSP27. When HSAEC cells are pre-treated with SB 203580 for 2 h, there is a significant decrease in the entry of Junin strain Romero pseudovirus with a dose–response (**Figure 2A**). The percent of infected cells pre-treated with SB 203580 for 2 h and infected with CD1 were also significantly decreased at the 2 highest concentrations, and there was a dose–response (**Figure 2B**). Similar results were found with both the Romero pseudovirus and CD1, thus the data indicate that the inhibition may not be a strain-dependent phenomenon and phosphorylated p38 MAPK may play a role in virus entry or in the early stages of replication of Junin viruses.

**Figure 3:**
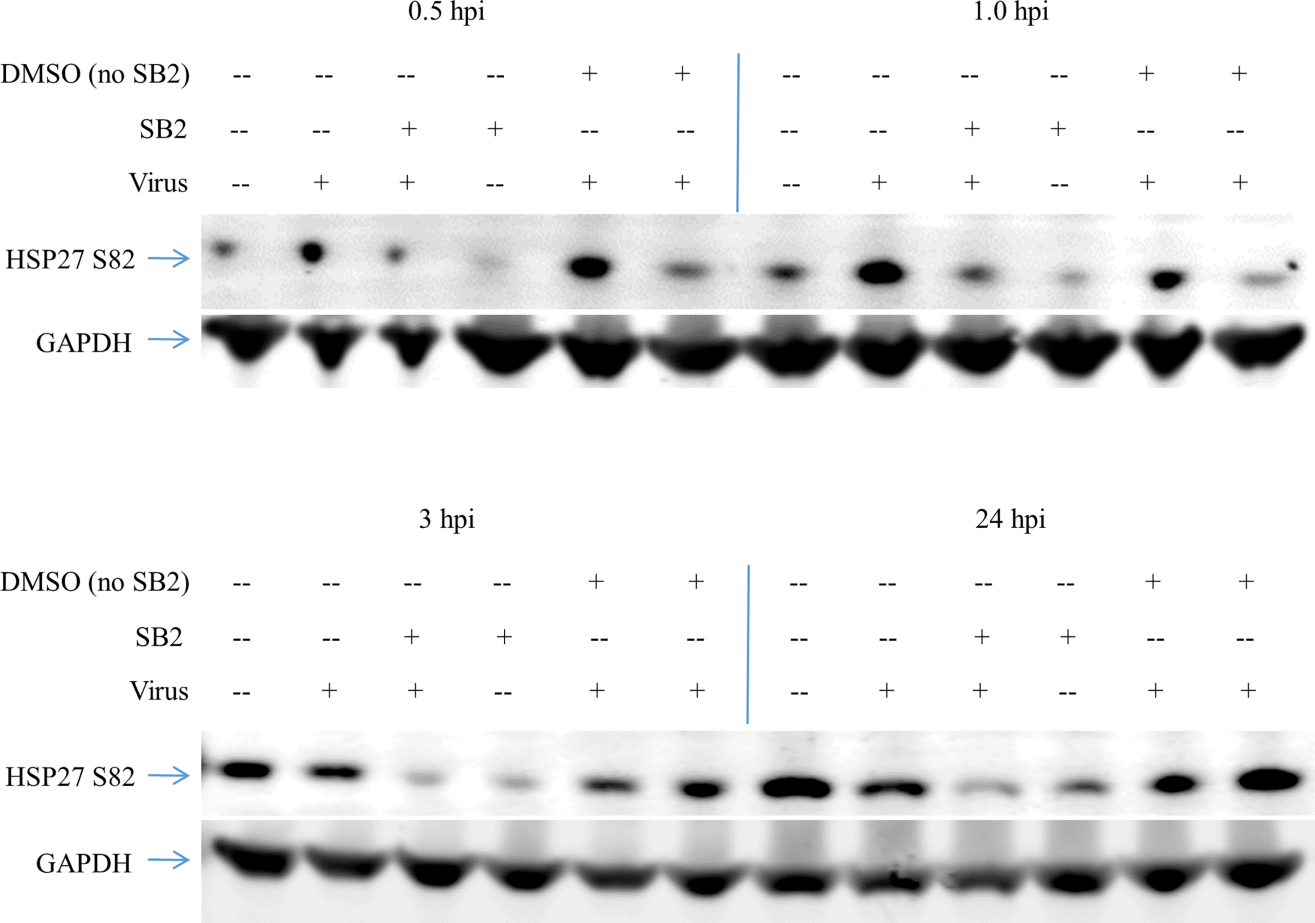
HSP27 phosphorylation upon JUNV infection results from p38 MAPK activity. HSAEC cells were pretreated with 40 nM SB 203580, or the equivalent concentration of DMSO, or media alone for 2 h, then infected with CD1 or mock infected in duplicate. At 0.5–24 h the cells were lysed, and then the duplicates were pooled and probed for HSP27 S82 and GAPDH by Western blot. One representative of two experiments is shown.

HSP27 can be phosphorylated by mitogen-activated protein kinase-activated kinase 2 and 3 (MK2/3), which are themselves activated through the p38 MAPK, or the extracellular signal-regulated protein kinase (ERK) signaling pathways, as well as Protein Kinase D. To determine if the phosphorylation of HSP27 is downstream of the p38 MAPK activation we measured the effect of SB 203580 on HSP27 phosphorylation upon CD1 infection (**Figure 3**). HSAEC cells were pretreated with SB 203580, the equivalent DMSO concentration, or media alone and either infected with CD1 (Virus, SB2 + Virus, DMSO + Virus) or media without virus was added (Mock, SB2, DMSO). The cells were then lysed at 0.5, 1, 3, or 24 h post infection and analyzed by Western Blot. In virus infected cells that were mock treated or DMSO treated there was an increase in HSP27 S82 at both 0.5 and 1 h post infection, with a much larger response when cells are treated with DMSO and infected with CD1. DMSO alone, without virus, also increased HSP27 phosphorylation over mock at the 0.5 h timepoint, and the effect was lost by1 h. Even though the SB 203580 treated cells were treated with the equivalent DMSO concentration, and there was a slight increase in phosphorylation of HSP27 with SB 203580 treatment alone at 0.5 h, the SB 203580 treated and virus infected cells had no increase in HSP27 S82 at any timepoint. The data suggests that the phosphorylation of HSP27 at S82 is downstream of the p38 MAPK pathway upon JUNV infection.

### Knockdown of p38 MAPK and HSP27 Decreases JUNV Infection in HSAEC Cells

**Figure 4:**
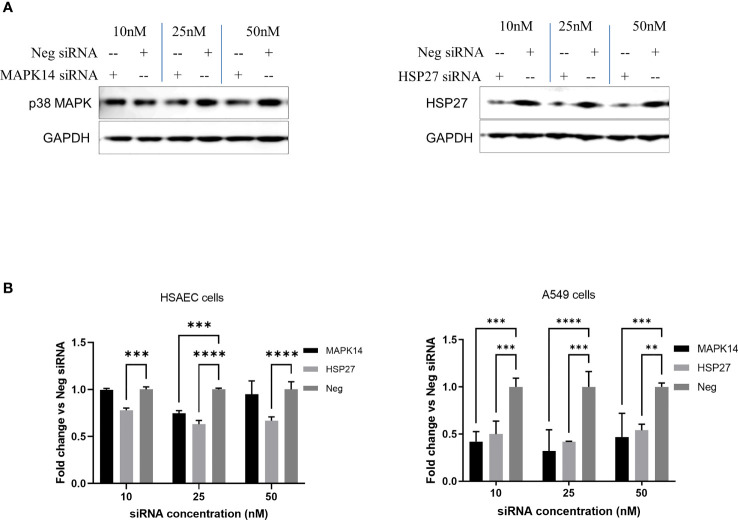
siRNA knockdown of p38 MAPK and HSP27 decrease JUNV infection. HSAEC and A549 cells in a 96-well plate were transfected with 10–50 nM of siRNA with DharmaFECT 1 transfection reagent in triplicate, HSAEC cells were transfected in duplicate plates. **(A)** Forty-eight hours post transfection HSAEC cells in one 96-well plate were lysed for Western blot analysis, 3 wells per condition were combined per lane to assess the level of protein knockdown. **(B)** Another 96-well plate per cell line was infected with JUNV CD1 with an MOI of 1, 24-hours post infection the JUNV nucleocapsid was stained with a mouse monoclonal anti-N antibody and detected with an Alexafluor-488 conjugated secondary. The signal was measured by high content imaging, data shown were triplicate biological samples in a single experiment, and these are representative of three experiments for HSAEC cells and one experiment for A549 cells. **p=0.0019, ***p<0.001, ****p<0.0001.

In addition to treatment with SB 203580, we examine the effect of the knockdown of p38 MAPK or HSP27 by specific siRNAs on CD1 replication in cells. HSAEC cells were transfected with 10–50 nM of siRNA specific for p38 MAPK (MAPK14), HSP27, or a non-specific control siRNA (Neg) and the effect on JUNV infection with CD1 was examined by high content imaging (**Figure 4B**). Both specific siRNAs decreased the target proteins as determined by Western blot analysis (**Figure 4A**). The siRNA for HSP27 significantly decreased CD1 infection with all siRNA concentrations tested. The siRNA for p38 MAPK (MAPK14) only had a significant effect on CD1 with 25 nM (**Figure 4B** **)**. We also examined the effect of the siRNA knockdown of p38 MAPK or HSP27 in another lung epithelial cell line, A549 (**Figure 4B**). All siRNA concentrations targeting both proteins significantly reduced CD1 infection in A549 cells.

## Discussion

We compared a vaccine strain of JUNV (CD1) to the most parental pathogenic virulent strain available (XJ13) and identified both shared alterations in the cellular response to infection and responses that were divergent between these strains. As shown in **Table 1**, most of the cellular responses that were similar between the strains correspond to entry and early replication events, at the 1 h post-infection timepoint, and these had the highest fold-changes overall. Following the RPPA analysis, we focused on the early entry events for verification and also explored the role of HSP27 and p38 MAPK in JUNV replication. Both HSP27 and NFκB are downstream of p38 MAPK signaling, and all three proteins are phosphorylated within 1 h of infection with both JUNV strains. NFκB rapidly responds to stimuli, such as IFNβ, thus it is possible that the transient NFκB response with JUNV at 1 h post infection may be due to IFNβ or other cytokines in the cell culture derived viral supernatant, but this may also result from a direct response of virus entry. Follow up studies should be performed to distinguish if the NFκB response is a direct response to virus entry. The specific role of HSP27 S82 in JUNV replication requires further investigation. Hsp27 is a chaperone protein that has multiple functions depending on the phosphorylation or oligomeric state of the protein. Unphosphorylated Hsp27 is primarily oligomerized and binds and transfers aggregation-prone proteins to the 20S proteasome for degradation. In addition, it has been shown to prevent cytochrome c release from mitochondria and bind to caspase 9 thereby inhibiting apoptosis (Garrido et al., 1999; Parcellier et al., 2006). Upon phosphorylation Hsp27 dissociates into lower order dimers or monomers species, which regulate actin stress fibers as well as prevent death-domain associated proteins interacting with FasL (Miron et al., 1991; Huot et al., 1995; Rogalla et al., 1999; Charette and Landry, 2000; Theriault et al., 2004; Arrigo et al., 2005). The early activation of p38 MAPK and subsequent phosphorylation of HSP 27, shown to occur at both 30 min and 1 h in **Figure 4**, may be advantageous to viral replication by preventing an early pro-apoptotic response upon virus entry and early replication events. JUNV enter the cells by attaching to cellular entry factors, including the transferrin receptor, presumably through clathrin-mediated endocytosis, and evidence points to fusion in the late endosome for the release of the ribonucleoproteins (Martinez et al., 2009). One group showed that actin dynamics have been shown to be important for JUNV infectivity, and the p38 MAPK and HSP27 interaction has been found to play a role in actin polymerization (Martinez et al., 2008). Both the phosphorylated and unphosphorylated forms may be beneficial to the virus during replication. Several viruses, such as influenza A virus and human respiratory syncytial virus, have also been shown to activate p38 MAPK within 10 min of infection through recognition of pattern recognition receptors by toll-like receptor 4 and MyD88 during virus entry (Marchant et al., 2010). An increase in p38 MAPK phosphorylation has been shown *in vitro* with Pichindé virus (PICHV), another arenavirus, and the timing and intensity differs between an attenuated and virulent strain of this virus (Bowick et al., 2007). There was a greater increase of p38 MAPK 1 day post infection (dpi) with attenuated PICHV strain in comparison to the virulent strain, and the inverse late in infection (6 dpi). Rajaiya et al. suggest that HSP27, p38 MAPK, and NFκB-p65 function as a signalosome during viral infection (Rajaiya et al., 2012). Through siRNA knockdown we cannot distinguish if the total decrease in p38 MAPK and HSP27 was detrimental to viral replication or if this was due to the decrease in the phosphorylated form. However, the siRNA knockdown results (**Figure 4**) combined with the SB 203580 (**Figure 2**) results suggests that the phosphorylation of HSP27 is downstream of p38 MAPK following JUNV infection, and this interaction plays a role in JUNV replication. Given that the phosphorylation of p38 MAPK and HSP27 occur within 30 min to 1 h (**Table 1** and **Figure 3**) and this phosphorylation is absent by 3 h and continues through 24 h post infection indicates that this pathway is involved in early replication events for JUNV. These two proteins may play other crucial roles in JUNV replication, and the reduction of these proteins through siRNA knockdown cannot distinguish between these other possible roles in replication. Future exploration into the specific role that the p38 MAPK/HSP27 pathway plays in JUNV replication is needed, as the mechanism was not determined in this work. SB 203580 was found to be protective against Dengue virus *in vivo*, the finding that this compound reduces JUNV infection *in vitro* (**Figure 2**), warrants further exploration of this compound, or other compounds targeting p38 MAPK activity, as a potential therapeutics (Sreekanth et al., 2016).

Not surprisingly, the majority of the divergent responses to the virus strains occurred mid-to-late in the replication cycle, 8–24 h post infection. Four modulations were identified that were unique to CD1. Three of the four are involved in the antiviral response of cells, and the modulations in CD1 may allow for an increased antiviral response to CD1 in comparison to XJ13. β-catenin is active in the unphosphorylated form, once hyper phosphorylated (S33, S37, T41, and Ser45) it is ubiquitinated and degraded (Aberle et al., 1997). Upregulation of the WNT/β-catenin pathway is associated with increased inflammation with several viruses, to include infections with SARS-CoV-2 (Choi et al., 2020), herpes simplex virus type 1 (HSV-1) (Harrison and Jones, 2021), and β-catenin is suppressed by influenza A virus (Hillesheim et al., 2014). Our results indicate that the hyperphosphorylated form of β-catenin is reduced in CD1 but not XJ13, which suggests that there may be more active β-catenin in response to CD1 infection which may result in increased inflammation. The reduction in hyperphosphorylated of β-catenin with CD1 is subtle here though. Phosphorylation of Smad 1/5/9 was only increased in the attenuated strain of JUNV at the 24-hour timepoint; in contrast, modulation of Smad signaling was found to be increased at mid-late replication timepoints (9 and 18 h post infection) with both an attenuated and virulent strain of Rift valley fever virus (de la Fuente et al., 2018). Smad 1/5/9 is a transcription factor that is dependent on bone morphogenic protein and transforming growth factor-beta mediated signaling, and Smad1 binds to multiple genes associated with the Type I IFN response (Eddowes et al., 2019). Suppressor of cytokine signaling 1 (SOCS1) is reduced in CD1 at the 24-hour timepoint, and SOCS1 is a repressor of type I signaling and a negative regulator of toll-like receptor and cytokine-induced signaling (Yoshimura et al., 2005; Fujimoto and Naka, 2010; Yan et al., 2019). Together these results may indicate a mechanism by which there would be an early increase in the inflammatory response in CD1 in comparison to XJ13.

Only one modulated protein was shared at the late timepoint between the two JUNV strains. Although AKT was modulated with both viruses in the mid to late timepoints, an increase of phosphorylation at T308 was only found with XJ13, followed by a decrease in phosphorylation at 24 h posts infection. AKT phosphorylation is suppressed following ER stress (Yung et al., 2011), and accumulation of the JUNV glycoproteins have been shown to induce ER stress (Manning et al., 2017; Manning et al., 2020). Yung et al. found that during ER stress phosphorylation at T308 is suppressed, but S473 is increased (Yung et al., 2011), We did not see an increase in S473 with our timepoints, but our 24-hour timepoint may not have been late enough to see this effect as Manning et al. found that glycoprotein aggregation increases significantly after 24 h (Manning et al., 2020). Linero et al. showed that JUNV strain XJCl3 induced phosphorylation of AKT at S473 within 30 min of infection in several cell lines, but the latest timepoint in that study was 240 min post infection (Linero and Scolaro, 2009). AKT has hundreds of reported substrates, controls pro-growth and pro-survival activities, and is under the control of negative feedback loops (Datta et al., 1997; Lopez-Carballo et al., 2002; Potter et al., 2002).

Several modulations were unique to strain XJ13 mid and late-infection: ASK1 (S83), PKCΔ (T505), and MPO. One activity of AKT is the negative regulation of ASK1 by phosphorylating it at S83. The phosphorylation of AKT T308 at the 8-hour timepoint with XJ13 coincides with the phosphorylation of ASK1 at S83, and both were dephosphorylated at the 24-hour timepoint with XJ13. ASK1 is involved in the apoptotic pathway and is negatively regulated through phosphorylation. The significant phosphorylation of ASK1 early would inactivate it and prevent it from potentially activating the JNK pathway; the result may suppress an apoptotic response early in virus replication. PKCΔ is a regulator of the inflammatory response, and human immunodeficiency virus, influenza virus, and herpesviruses modulate PKCΔ at various stages of their replication cycles (Sieczkarski et al., 2003; Leach and Roller, 2010; Contreras et al., 2012).

In addition to shedding some light as to some of the pathways changes that result from JUNV entry into host cells, these studies highlight a few possible targets for further exploration for antivirals. For example, the p38 MAPK small molecule inhibitor SB 203580 has been shown to be protective in mouse studies for Dengue virus, showing the utility of targeting some of these pathways for potential treatments (Sreekanth et al., 2016). Further work is also needed to examine the mechanism by which the pathogenic strain of JUNV may augment the antiviral and pro-survival responses in host cells, while the attenuated strain does not. Numerous key players in antiviral and pro-survival pathways were highlighted in this work, and the differences in these responses between the two strains should be explored further to answer some of these questions.

## Data Availability Statement

The original contributions presented in the study are included in the article/**Supplementary Material**. Further inquiries can be directed to the corresponding author.

## Author Contributions

AG, CF, LA, JT, and RM performed the experiments. CC processed and analyzed the RPPA data. AG, CJF, and RM analyzed all other data. AG, CS, BB, AN, CDF, and KKH designed the experiments. AG wrote the manuscript with revisions by CF, and RM. All authors listed have made a substantial, direct, and intellectual contribution to the work and approved it for publication.

## Funding

This work was funded through a Defense Threat Reduction Agency (DTRA) grant HDTRA1-13-1-0006. DTRA does not have any role in the design of the study and collection, analysis, and interpretation of data and nor in writing the manuscript.

## Author Disclaimer

Opinions, interpretations, conclusions, and recommendations are those of the author and are not necessarily endorsed by the U.S. Army.

## Conflict of Interest

Author CC is/was employed by DCE Consulting Inc. 

The remaining authors declare that the research was conducted in the absence of any commercial or financial relationships that could be construed as a potential conflict of interest.

## Publisher’s Note

All claims expressed in this article are solely those of the authors and do not necessarily represent those of their affiliated organizations, or those of the publisher, the editors and the reviewers. Any product that may be evaluated in this article, or claim that may be made by its manufacturer, is not guaranteed or endorsed by the publisher.

## Supplementary Material

Supplementary Figure 1 Optimizing infection of HSAEC cells with JUNV XJ13 and CD1, and experimental layout. **(A)** HSAEC cells in a 96 well plate were infected with either JUNV strain XJ13 or strain CD1 from an MOI of 0-34 or 0-39 respectively, 3 wells per MOI. After 24 hours the cells were fixed in 10% buffered formalin and stained for JUNV NP and analyzed by HCI to calculate the % positive cells.

Supplementary Table 1 All RPPA results. RPPA results for JUNV XJ13 (XJ) and Candid#1 (CD) compared to mock infected cells. Changes highlighted in yellow (decreased phosphorylation) or blue (increased phosphorylation) have at least a 1.2 fold change and a p ≤ 0.05 as measured by a t-test.
