# Supplementary figures and images for: Junin Virus Activates p38 MAPK and HSP27 Upon Entry
Source: Front Cell Infect Microbiol. 2022 Apr 7;12:798978. doi: 10.3389/fcimb.2022.798978 (PMC9022028; doi:10.3389/fcimb.2022.798978)

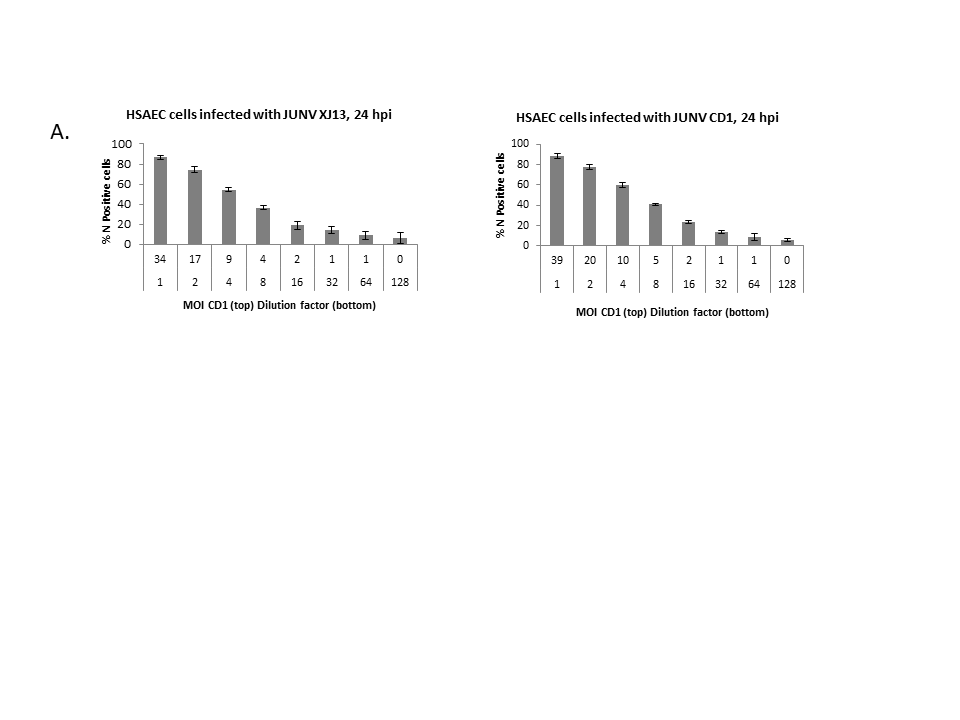

Supplement: Supplementary Figure 1 — Optimizing infection of HSAEC cells with JUNV XJ13 and CD1, and experimental layout. (A) HSAEC cells in a 96 well plate were infected with either JUNV strain XJ13 or strain CD1 from an MOI of 0-34 or 0-39 respectively, 3 wells per MOI. After 24 hours the cells were fixed in 10% buffered formalin and stained for JUNV NP and analyzed by HCI to calculate the % positive cells. [file Image_1.tif]
